# New adaptive laboratory evolution database highlights the need for consolidating directed evolution data

**DOI:** 10.1093/synbio/ysz004

**Published:** 2019-02-04

**Authors:** Daniel Bojar

**Affiliations:** 4058 Basel, Switzerland

Directed evolution, honored by the 2018 chemistry Nobel prize, uses mutagenesis and selective pressure to drive proteins towards the development of new and improved functionalities. A closely related approach, known as Adaptive Laboratory Evolution (ALE) applies similar principles to optimize whole strains for desired growth conditions.^1^ While these approaches have resulted in considerable advances in the realms of enzyme and strain optimization, they remain isolated endeavors. A new resource, the Adaptive Laboratory Evolution Database (ALEdb),^2^ is a first step towards concentrating efforts to harness the power of evolution for new and improved phenotypes.

In ALE experiments, organisms such as *Escherichia coli* or *Saccharomyces cerevisiae* are grown for extended periods of time under specified culture conditions to enable the acquisition of advantageous or adaptive mutations. Developed as a web-based platform hosted by the University of California, San Diego, ALEdb was launched with over 11 000 mutations and the corresponding culture conditions gathered from 11 publications and is built to be expanded by further studies.

The mutations stored at ALEdb are characterized by the genome position, the affected gene, the type of mutation, and the cell culture conditions used in the ALE experiment. Data from different studies can be exported and analyzed from ALEdb by data mining for trends or patterns in mutations to generalize and advance the pursuit of improved and novel functionalities. Additionally, ALEdb serves as a knowledge database where scientists can functionally characterize new mutations discovered by comparing them to records stored in ALEdb.

To showcase the utility of ALEdb, the authors investigated mutation type distributions from already cataloged studies. Single nucleotide polymorphisms (SNPs) were found to be the most frequent. The authors argue that this trend suggests the importance of SNPs for adaptive evolution, though it is hard to support this claim without taking into account the baseline frequency of different mutation types.

In the context of synthetic biology, a concerted database of adaptive mutations like ALEdb could help guide the rational design of new and improved functionalities. However, ALEdb currently catalogs ALE-derived mutations only in naturally occurring genes. Expanding the database to include variations resulting from directed evolution of standard genetic parts that may not be native to the host organism would greatly increase its influence.

Additionally, databases such as ALEdb are dependent on continued submissions. Since it was first described in early October, the publication count on ALEdb has expanded from 11 to 33. This early momentum is a promising sign, but it is unclear how the authors envision the assured spread and maintenance of ALEdb.

Potential ways forward could include a pledge by journals to require data submission—similar to the way the protein data bank PDB (Protein Data Bank) handles 3D protein structures—or linkage with other established databases, such as the systems biology Pseudomonas species database, SYSTOMONAS. This database partnership might create additional synergy, as a greater breadth of data would be accompanied by a greater depth, facilitating new kinds of analyses.

If nurtured and expanded, ALEdb could prove a useful platform for exploring and utilizing general principles of evolution. In the meantime, the announced launch of ALEdb should stimulate discussions about the importance of consolidating results from adaptive laboratory and directed evolution experiments. Under the right conditions, ALEdb or a similar database could evolve to include mutagenesis conditions and non-native genes, ultimately incorporating the full gamut of directed evolution data.
